# Modulation of Intestinal Inflammation by Yeasts and Cell Wall Extracts: Strain Dependence and Unexpected Anti-Inflammatory Role of Glucan Fractions

**DOI:** 10.1371/journal.pone.0040648

**Published:** 2012-07-27

**Authors:** Samir Jawhara, Khalid Habib, François Maggiotto, Georges Pignede, Pascal Vandekerckove, Emmanuel Maes, Laurent Dubuquoy, Thierry Fontaine, Yann Guerardel, Daniel Poulain

**Affiliations:** 1 Université Lille Nord de France, Lille, France; 2 UDSL, Lille, France; 3 INSERM U995, Lille, France; 4 Service de Parasitologie Mycologie, Pole de Biologie Pathologie Génétique, Lille, France; 5 CHRU Lille, Lille, France; 6 Université de Lille 1, Unité de Glycobiologie Structurale et Fonctionnelle, UGSF, Villeneuve d'Ascq, France; 7 CNRS, UMR 8576, Villeneuve d'Ascq, France; 8 Lesaffre International, Division R&D, Marcq-en-Baroeul, France; 9 Unité des Aspergillus, Institut Pasteur, Paris, France; Institut National de la Santé et de la Recherche Médicale U 872, France

## Abstract

Yeasts and their glycan components can have a beneficial or adverse effect on intestinal inflammation. Previous research has shown that the presence of *Saccharomyces cerevisiae* var. *boulardii* (Sb) reduces intestinal inflammation and colonization by *Candida albicans*. The aim of this study was to identify dietary yeasts, which have comparable effects to the anti-*C. albicans* and anti-inflammatory properties of Sb and to assess the capabilities of yeast cell wall components to modulate intestinal inflammation. Mice received a single oral challenge of *C. albicans* and were then given 1.5% dextran-sulphate-sodium (DSS) for 2 weeks followed by a 3-day restitution period. *S. cerevisiae* strains (Sb, Sc1 to Sc4), as well as mannoprotein (MP) and β-glucan crude fractions prepared from Sc2 and highly purified β-glucans prepared from *C. albicans* were used in this curative model, starting 3 days after *C. albicans* challenge. Mice were assessed for the clinical, histological and inflammatory responses related to DSS administration. Strain Sc1-1 gave the same level of protection against *C. albicans* as Sb when assessed by mortality, clinical scores, colonization levels, reduction of TNFα and increase in IL-10 transcription. When Sc1-1 was compared with the other *S. cerevisiae* strains, the preparation process had a strong influence on biological activity. Interestingly, some *S. cerevisiae* strains dramatically increased mortality and clinical scores. Strain Sc4 and MP fraction favoured *C. albicans* colonization and inflammation, whereas β-glucan fraction was protective against both. Surprisingly, purified β-glucans from *C. albicans* had the same protective effect. Thus, some yeasts appear to be strong modulators of intestinal inflammation. These effects are dependent on the strain, species, preparation process and cell wall fraction. It was striking that β-glucan fractions or pure β-glucans from *C. albicans* displayed the most potent anti-inflammatory effect in the DSS model.

## Introduction

Probiotics are a popular alternative to antibiotics [1]. The positive effects of probiotics on humans and animals result either from a direct nutritional effect or a health effect, with probiotics acting as bioregulators of the intestinal microflora and reinforcing the host's natural defences [2].

*Saccharomyces cerevisiae* var. *boulardii* (Sb) is described as a biotherapeutic agent in the clinical literature and is reported to be efficacious in the prevention of antibiotic-associated diarrhoea and colitis in humans [3], [4]. Orally administered Sb demonstrated clinical and experimental effectiveness in gastrointestinal diseases through modulation of host cell signalling pathways implicated in the pro-inflammatory response such as IL-1β and TNF-α. Sb exerts a trophic effect that restores intestinal homeostasis and activates expression of peroxisome proliferator–activated receptor-gamma which protects against gut inflammation [5].

It has recently been reported that Sb decreases inflammation and intestinal colonization by *C. albicans* in a BALB/c mouse model of colitis induced by dextran-sulphate-sodium (DSS) [6]. Interestingly, parallel studies indicated that Sb reduces *C. albicans* adhesion to human intestinal cell lines and decreases pro-inflammatory cytokine mRNA levels in response to *C. albicans* infection [7], [8].

Sb is generally administered as a lyophilized powder [9] and its use as a food additive has only been reported in a number of cases such as in the fermentation of raw vegetable materials [10] and incorporation into commercial yoghurts [11].Taxonomic studies indicate that Sb should be considered as a *Saccharomyces cerevisiae* strain [12], [13]. This leads to the question “do other strains of *S. cerevisiae* also possess probiotic properties?” [14].

In the present study, low doses of DSS were administered to mice for 2 weeks to induce colonic inflammation and promote the establishment of *C. albicans* colonization, followed by a 3-day restitution period. Either *S. cerevisiae* strains or glycan fractions were then administered daily by oral gavage for 2 weeks, starting 3 days after the *C. albicans* challenge, in order to assess their curative effects on both colonic inflammation and acceleration of colonic epithelial restoration. Using the DSS mouse model each dietary yeast was found to have its own effect on colitis and *C. albicans* colonization. The impact of orally administered glycan fractions extracted from *S. cerevisiae* that may reverse the adverse effects of DSS and *C. albicans*, and the biological activity of soluble β-glucan isolated from the *C. albicans* cell wall, were then investigated in this DSS mouse model.

## Results

### Comparison of the probiotic potential of *S. cerevisiae* var. *boulardii* and *S. cerevisiae* 1-1 strain

*S. cerevisiae* 1-1 strain (Sc1-1) was selected from our collection as having previously exhibited a probiotic effect. This yeast is prepared as an active dry yeast so that it can react quickly to its environment (Table 1). As Sc1-1 was comparable *in vitro* to Sb in terms of decreasing growth and germ tube formation by *C. albicans* (data not shown), the aim was to compare the Sc1-1 and Sb strains for their ability to reduce *C. albicans* colonization and intestinal inflammation.

**Table 1:** Yeast strains used in the investigation.

Strains	Reference	Description	Comments	Source
*C. albicans*	SC5314	Pathogenic strain		[64]
*S. cerevisiae* var. *boulardii* **Sb**	CNCM I-3799	Probiotic strain^(Patented)^	Instant dried yeast (vermicelli)	Lesaffre Yeast Collection
*S. cerevisiae* ***Sc1-1***	CNCM I-3856	Probiotic strain^(Patented)^	Active dried yeast (gastro-resistant spherules)	Lesaffre
*S. cerevisiae* ***Sc1-2***	CNCM I-3856	Probiotic strain^(Patented)^	Instant dried yeast (vermicelli)	Lesaffre
*S. cerevisiae* ***Sc2*** ***Sc2 MP*** ***Sc2 GP***	LYSC318E	Autolytic properties, Protein rich strainUsed for preparation of MPs and GPs cell wall fractions	Cream	Lesaffre
*S. cerevisiae* ***Sc3***	LYSC3-5b	Osmotolerant strain	Instant dried yeast (vermicelli)	Lesaffre
*S. cerevisiae* ***Sc4***	LYSC17-82A	Thermosensitive mutant	Instant dried yeast (vermicelli)	Lesaffre

**Figure 1:**
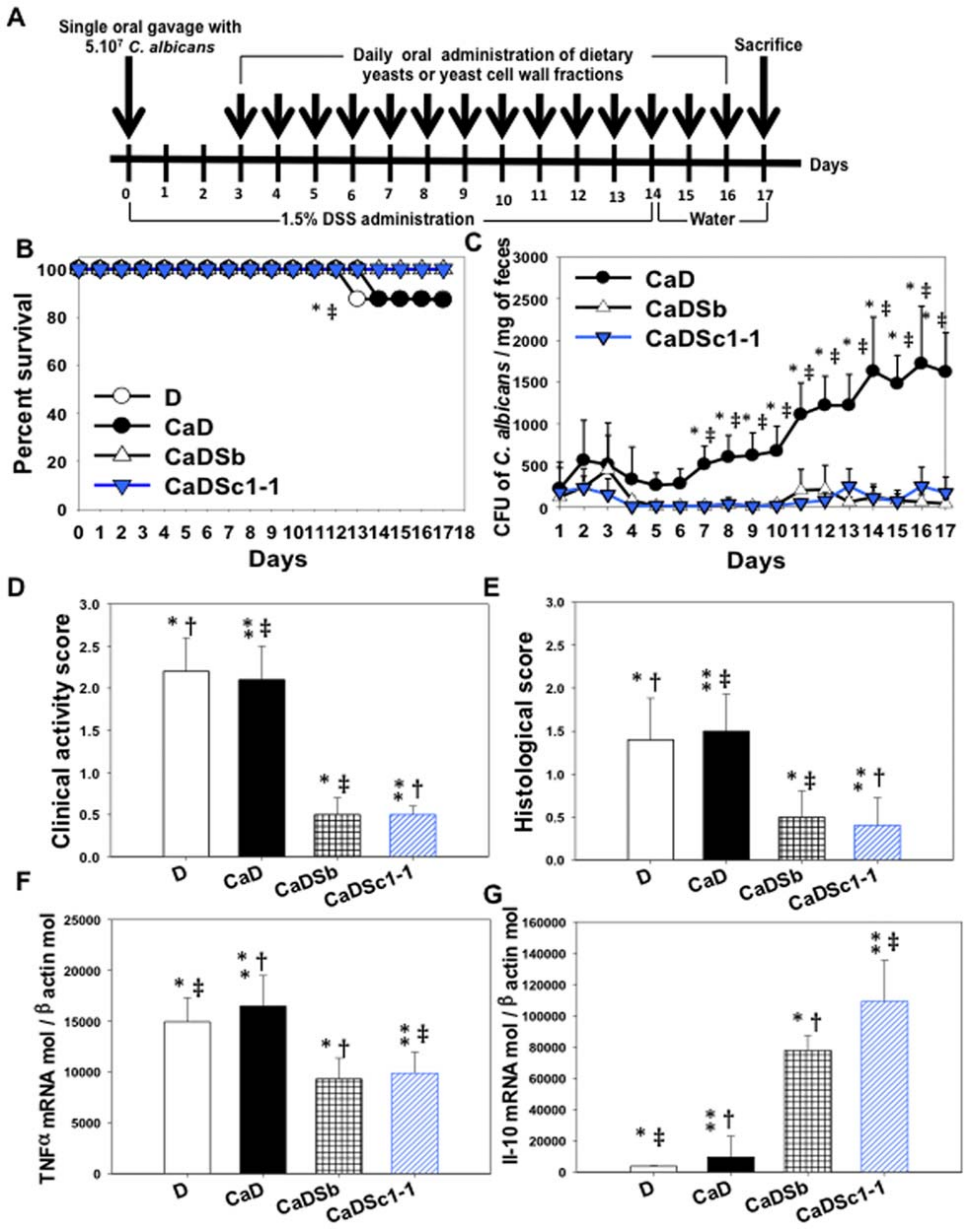
Comparison of the probiotic potential of *S. cerevisiae* var. *boulardii* and *S. cerevisiae* 1-1 strain. (**A**)** Experimental design**. Low doses of DSS were used for 2 weeks followed by a 3-day restitution period. Either *S. cerevisiae* strains or β-glucan fractions were inoculated daily by oral gavage starting 3 days after *C. albicans* challenge. Three days post-*C. albicans* challenge was chosen to start *S. cerevisiae* treatment as both *Candida* colonization and colonic inflammation are well established at this time point. (**B**)** Percentage survival of mice**. The results are shown as percent survival from the time of *C. albicans* challenge and DSS treatment. The survival data were significantly different by the log-rank test. A total of 140 mice were divided into five control groups: water (n = 16 mice), Ca (n = 16 mice), Sb (n = 16 mice), Sc1-1 (n = 16 mice) and DSS alone (n = 16 mice), and three experimental groups: CaD (n = 20 mice), CaDSb (n = 20 mice), and CaDSc1-1 (n = 20 mice). (*P<0.05 for CaDSb mice vs. CaD mice; and ‡*P*<0.05 for CaDSc1-1 mice vs. CaD mice.) (**C**)** Number of** ***C. albicans*** **colony forming units recovered from stools**. Each data set represents the mean values of 10 mice/group. (*P<0.05 for CaDSb mice vs. CaD mice; and ‡*P*<0.05 for CaDSc1-1 mice vs. CaD mice.) (**D**)** Clinical analysis of DSS-induced colitis in mice**. Both *S. cerevisiae* var. *boulardii* and *S. cerevisiae* Sc1-1 significantly reduced the clinical score. (*P<0.05 for CaDSb mice vs. D mice; ‡*P*<0.05 for CaDSb mice vs. CaD mice; †*P*<0.05 for CaDSc1-1 mice vs. D mice; and **P<0.05 for CaDSc1-1 mice vs. CaD mice.) (**E**)** Histological score**. The histological score was determined by two independent, blinded examiners (degree of inflammation: 0, no changes, to 6, extensive cell infiltration and tissue damage). The histological score increased in both DSS and CaDSS groups. The histological score was significantly lower in both CaDSb and CaDSc1-1 groups when compared to CaDSS and DSS-treated groups. (*P<0.05 for CaDSb mice vs. D mice; ‡*P*<0.05 for CaDSb mice vs. CaD mice; †*P*<0.05 for CaDSc1-1 mice vs. D mice; and **P<0.05 for CaDSc1-1 mice vs. CaD mice.) (**F and G**)** Relative expression levels, determined by real-time quantitative PCR, of TNF-** ***α*** **and IL-10 mRNA in the colon**. Data are expressed as the mean ± SE of five mice in each group. (*P<0.05 for CaDSb mice vs. D mice; †*P*<0.05 for CaDSb mice vs. CaD mice; ‡*P*<0.05 for CaDSc1-1 mice vs. D mice; and **P<0.05 for CaDSc1-1 mice vs. CaD mice.)

The model used is summarized in Fig. 1A and can be defined as “curative”. In this model, a single dose of *C. albicans* was administered to mice receiving DSS. Either the Sb or Sc1-1 strain was given 3 days later when *C. albicans* colonization was established. Low mortality was observed in mice that received DSS or DSS+*C. albicans* whereas none of the mice given the same regimen plus *S. cerevisiae* died (Fig. 1B).

Concerning *C. albicans* colonization in mice that received DSS (Fig. 1C), the number of colony-forming units (CFUs) in stools gradually increased from the day of *C. albicans* administration. From *S. cerevisiae* administration on day 4 to the endpoint, a dramatic reduction in number of *C. albicans* CFUs was observed. No difference was observed between the two *S. cerevisiae* strains in their activity on *C. albicans* clearance (Fig. 1C).

**Figure 2:**
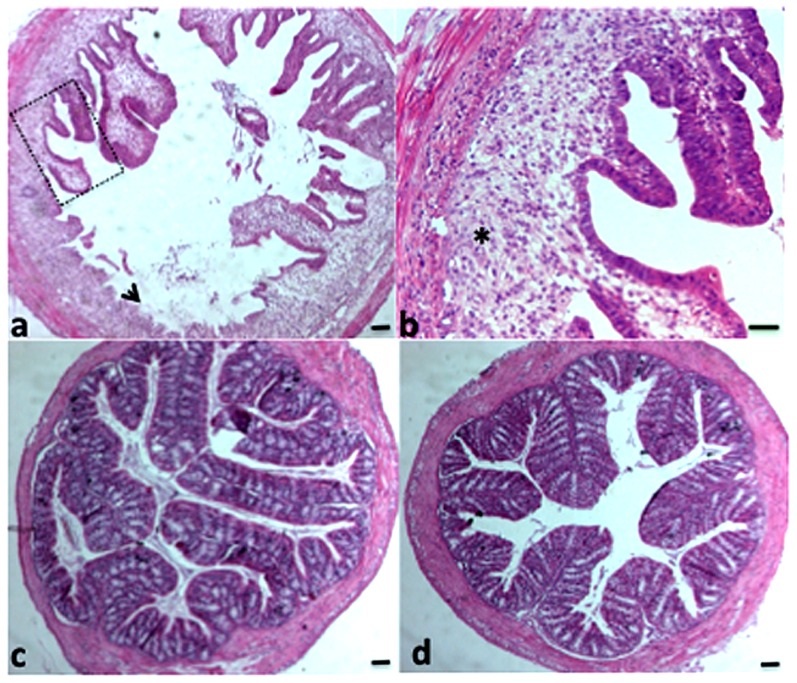
Histological analysis of DSS-induced colitis in mice. Colon sections (4 µm thick) were stained with haematoxylin/eosin. Panel (a and b) show colon sections from a mouse colonized with *C. albicans* and treated with DSS; panel (c) shows colon sections from a *C. albicans*+Sc1-1 DSS-treated mouse; and panel (d), colon sections from a *C. albicans*+Sb DSS-treated mouse. Colon sections from the *C albicans*+DSS-treated mouse showed an inflammatory cell infiltrate in colonic wall structures (arrow, asterisk). Colon sections from the mouse with DSS-induced colitis colonized with *C. albicans*+either Sb or Sc1-1 showed an attenuated inflammatory infiltrate in the submucosa with the presence of occasional leukocytes in the lamina propria of the mucosa (panels c and d). The scale bars represent 50 µm (panels a, c, and d) and 10 µm (panel b).

The cumulated clinical and histological scores (Fig. 1D–E) were higher in mice that received DSS or DSS+*C. albicans* whereas they were reduced significantly by the administration of either Sb or Sc1-1 (Fig. 1D–E). Both strains were equally effective in reducing intestinal inflammation assessed by these parameters (Fig. 2).

To analyze the possible mediators involved in the reduction of the inflammatory response to DSS and *C. albicans* in the colon following *S. cerevisiae* administration, we focused on levels of TNF-α and IL-10 mRNA as representative pro- and anti-inflammatory cytokines. As shown in Fig. 1F–G, administration of either Sb or Sc1-1 was associated with a significant reduction in TNF-α expression and increase in IL-10 production. No difference was observed between the two *S. cerevisiae* strains in their ability to redirect the inflammatory response.

### Analysis of the anti-*C. albicans* and anti-inflammatory properties of other *S. cerevisiae* strains with different preparation processes

As both Sc1-1 and Sb strains displayed identical beneficial effects in this specific model the Sc1-1 strain was used as the reference strain for probiotic activity. In this part of the study, the influence of yeast preparation procedure and other yeast strains was investigated.

From an initial screening involving 10 strains or preparation procedures, five representative examples of strain activities were selected and compared to Sc1-1. These consisted of strains Sc1-2, Sc2, Sc3 and Sc4.

**Figure 3:**
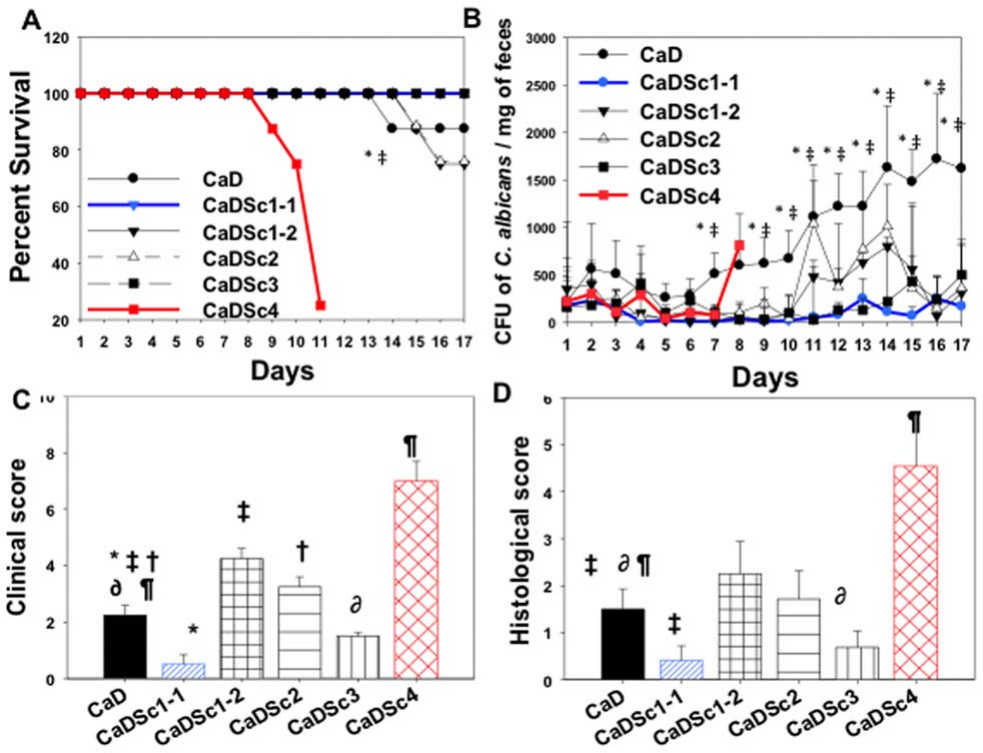
Assessment of biological effect of *S. cerevisiae* 1-1 versus other *S. cerevisiae* strains. (**A**)** Percentage survival of mice.** Results are shown as percent survival from the time of *C. albicans* challenge and DSS treatment. Three days after *C. albicans* challenge, mice were given one of five *S. cerevisiae* strains: Sc1-1, Sc1-2, Sc2, Sc3 or Sc4 for 2 weeks. The survival data were significantly different by the log-rank test. A total of 60 mice were divided into six experimental groups: CaD (n = 10 mice), CaDSc1-1 (n = 10 mice), CaDSc1-2 (n = 10 mice), CaDSc2 (n = 10 mice), CaDSc3 (n = 10 mice) and CaDSc4 (n = 10 mice). (*P<0.05 for CaDSc3 mice vs. CaD mice; and ‡*P*<0.05 for CaDSc1-1 mice vs. CaD mice.) (**B**)** Number of** ***C. albicans*** **colony forming units recovered from stools**. Each data set represents the mean values of eight mice per group. (*P<0.05 for CaDSc3 mice vs. CaD mice; and ‡*P*<0.05 for CaDSc1-1 mice vs. CaD mice.) (**C**)** Clinical analysis of DSS-induced colitis in mice**. Clinical score was determined by assessing weight loss, change in stool consistency and the presence of gross bleeding. The clinical score ranged from 0 to 8. (*P<0.05 for CaDSc1-1 mice vs. CaD mice; ‡*P*<0.05 for CaDSc1-2 mice vs. CaD mice; †*P*<0.05 for CaDSc2 mice vs. CaD mice; ∂*P*<0.05 for CaDSc3 mice vs. CaD mice; and ¶*P*<0.05 for CaDSc3 mice vs. CaD mice.) (**D**)** Histological score**. Mice were exposed to 1.5% DSS in drinking water for 14 days. Degree of inflammation: 0, no changes, to 6, extensive cell infiltration and tissue damage. In CaDSc1-1 and CaDSc3 groups, the histological score was significantly lower than that of CaD mice. Sc4 administration dramatically increased the histological score in *Candida* DSS-treated mice. (*P<0.05 for CaDSc1-1 mice vs. CaD mice; ‡*P*<0.05 for CaDSc1-2 mice vs. CaD mice; †*P*<0.05 for CaDSc2 mice vs. CaD mice; ∂*P*<0.05 for CaDSc3 mice vs. CaD mice; and ¶*P*<0.05 for CaDSc3 mice vs. CaD mice.)

**Figure 4:**
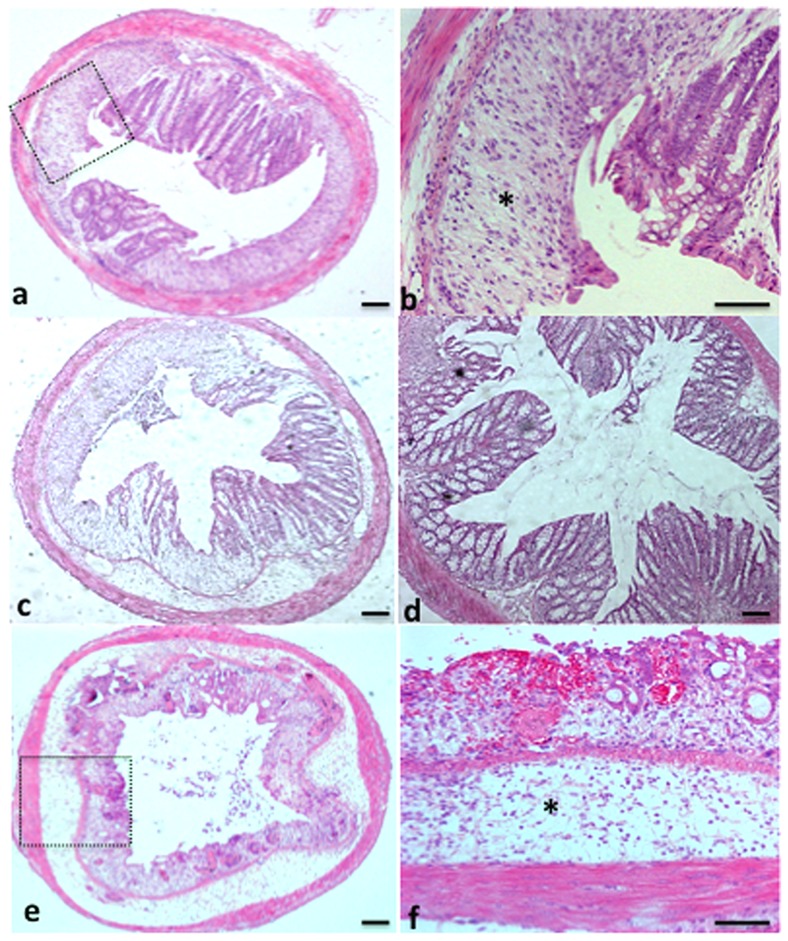
Histological analysis of DSS-induced colitis in mice. Panels (a), (c), (d) and (e) correspond to colon sections from CaDSc1-2, CaDSc2, CaDSc3 and CaDSc4 mice, respectively. The colon sections from CaDSc1-2, CaDSc2 and CaDSc4 mice showed an inflammatory cell infiltrate in colonic wall structures (asterisks, panels b and f) and tissue destruction with loss of both crypts and epithelial integrity (panels a, c and e). The colon sections from CaDSc3 revealed a lower inflammatory cell infiltrate in colonic wall structures and restoration of tissue destruction (panel D). The scale bars represent 50 µm (panels a, c, d and e) and 10 µm (panels b and f).

Sc1-1 showed important differences in mortality (Fig. 3A), ability to reduce *C. albicans* colonization (Fig. 3B) and a decrease in both histological and clinical scores (Fig. 3C–D). The Sc1-1 strain gave excellent results for all parameters, as did Sc3, which is used for its probiotic activities (Fig. 4) Interestingly, some of the beneficial effects induced by Sc1-1 were abolished when this strain was prepared as an instant dry yeast (Table 1). The most striking results were observed with the Sc4 strain, which did not display any particular effect on *C. albicans* colonization over 2 days compared to the other *S. cerevisiae* strains. However, the mice were extremely constipated, clinically inflamed and highly colonized with *C. albicans* starting from day 7. From this point on, we discontinued collecting faeces as the mice were becoming extremely ill. This yeast, which presents optimal growth at low temperatures, dramatically exacerbated both the clinical and histological scores and was associated with high mortality (around 80%). We also found that both *C. albicans* and Sc4 increased the staging of colitis in mice (Fig. 4). These high clinical and histological scores were associated with high numbers of *C. albicans* CFUs in different parts of the gut from moribund mice (data not shown).

Finally, Sc2, another industrial strain used for the production of yeast proteins, displayed an “intermediate” behaviour with reduced beneficial effects and slight worsening of mortality and clinical scores.

### Identification of the cell wall fractions supporting the beneficial and adverse effects on *C. albicans* colonization and inflammation

**Figure 5:**
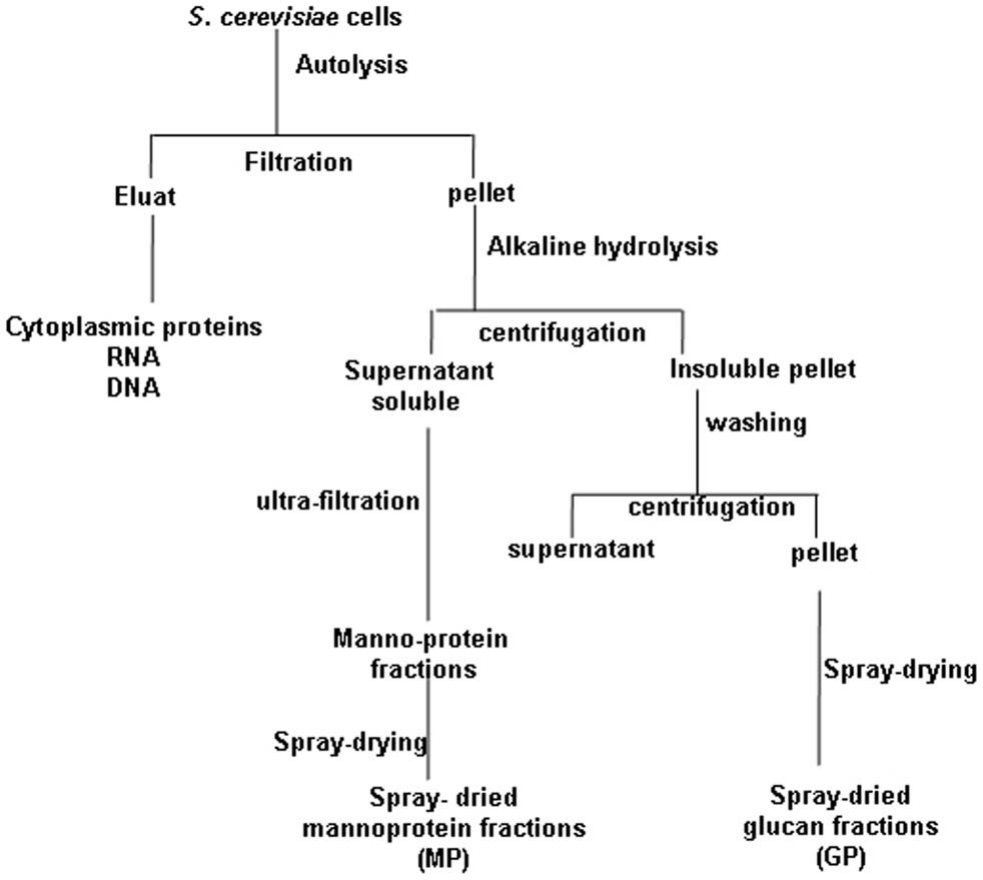
Schematic diagram of glycan preparation from *S. cerevisiae*.

For this purpose, Sc2 was used as this strain can be easily induced to undergo autolysis in order to prepare cell wall extracts including mannoprotein (MP) and β-glucan fractions (Fig. 5).

**Figure 6:**
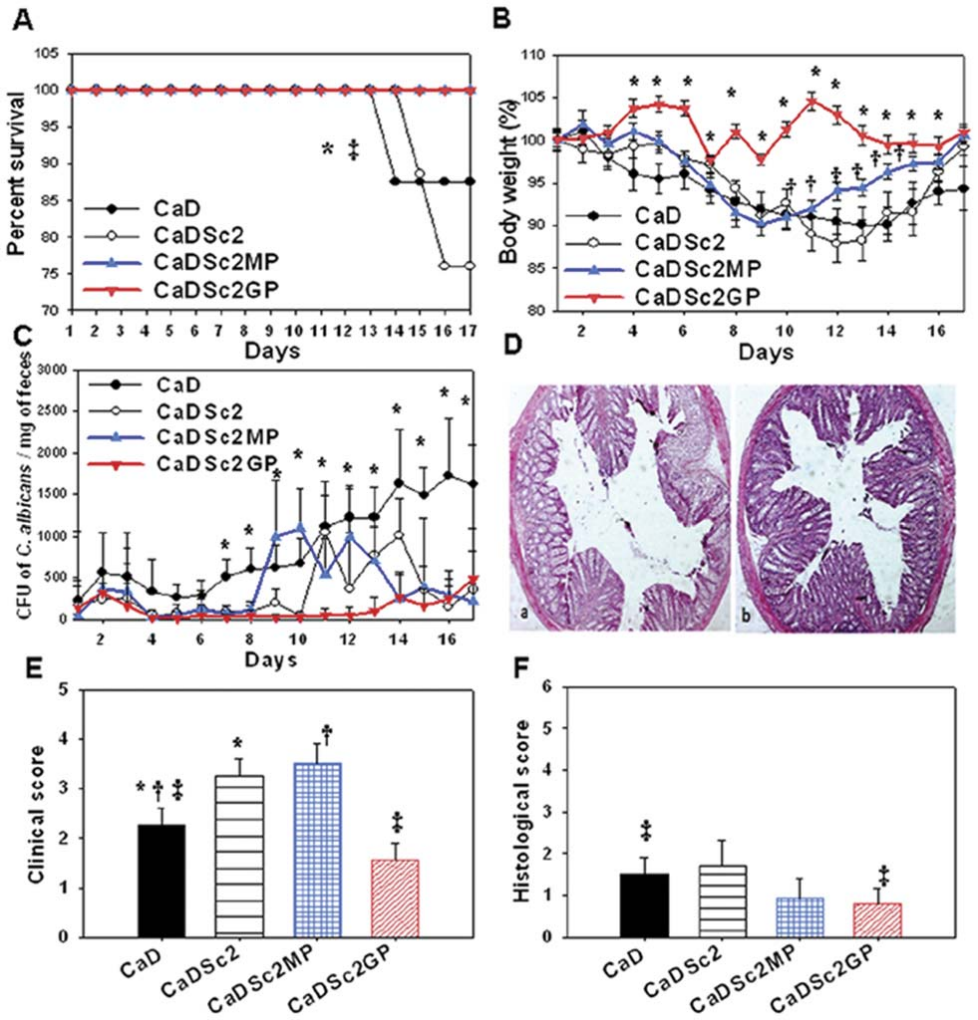
Effect of glycan fractions derived from *S. cerevisiae* on *Candida* DSS-treated mice. (**A**)** Percentage survival of mice**. The results are displayed as percent survival from the time of *C. albicans* challenge and DSS treatment. Three days after *C. albicans* challenge, mice were given either *S. cerevisiae* (Sc2), mannoproteins (Sc2MP) or β-glucan fractions (Sc2GP) for 2 weeks. The survival data were significantly different by the log-rank test. A total of 40 mice were divided into four experimental groups. (*P<0.05 for CaDSc2GP mice vs. CaD mice; and ‡*P*<0.05 for CaDSc2MP mice vs. CaD mice.) (**B**)** Difference in body weight loss in mice**. Each data set represents the mean value for each body weight. (*P<0.05 for CaDSc2GP mice vs. CaD mice, and †*P*<0.05 for CaDSc2MP mice vs. CaD mice.) (**C**)** Number of** ***C. albicans*** **colony forming units recovered from stools**. Each data set represents the mean value of 10 mice/group. (*P<0.05 for CaDSc2GP mice vs. CaD mice.) (**D**)** Histological analysis of DSS-induced colitis in mice**. Panel (a) shows a colon section from a *C. albicans*+mannoprotein DSS-treated mouse; panel (b) shows a colon section from a *C. albicans*+β-glucan DSS-treated mouse. Inflammatory cell infiltrates were insignificant in both CaDSc2MP and CaDSc2GP mice. (**E**)** Clinical analysis of DSS-induced colitis in mice**. Clinical score was determined by assessing weight loss, change in stool consistency and the presence of gross bleeding. The clinical score ranged from 0 to 8. (*P<0.05 for CaDSc2 mice vs. CaD mice; †*P*<0.05 for CaDSc2MP mice vs. CaD mice; and ‡*P*<0.05 for CaDSc2GP mice vs. CaD mice.) (**F**)** Histological score**. The histological score decreased significantly in CaDSc2GP mice. (*P<0.05 for CaDSc2 mice vs. CaD mice; †*P*<0.05 for CaDSc2MP mice vs. CaD mice; and ‡*P*<0.05 for CaDSc2GP mice vs. CaD mice.)

In contrast to living cells, none of the cell wall extracts induced mortality (Fig. 6A). However, the changes in body weight and clinical scores were worsened considerably by MP fractions while these parameters were ameliorated by β-glucan fractions (Fig. 6B, F). Notably, the MP fraction as well as the Sc2 strain induced an important loss in body weight up to day 9 that was correlated with its incapacity to control *C. albicans* colonization (Fig. 6C). By contrast, administration of β-glucan fraction maintained normal body weight, reduced inflammation scores and promoted *C. albicans* clearance (Fig. 6A–F). Although the body weight of mice receiving MP fractions started to increase from day 10, the clinical activity score was higher than that of mice receiving β-glucan fractions (Fig. 6E).

### Activity of the homologous *C. albicans* oligoglucoside fraction in the DSS mouse model

**Figure 7:**
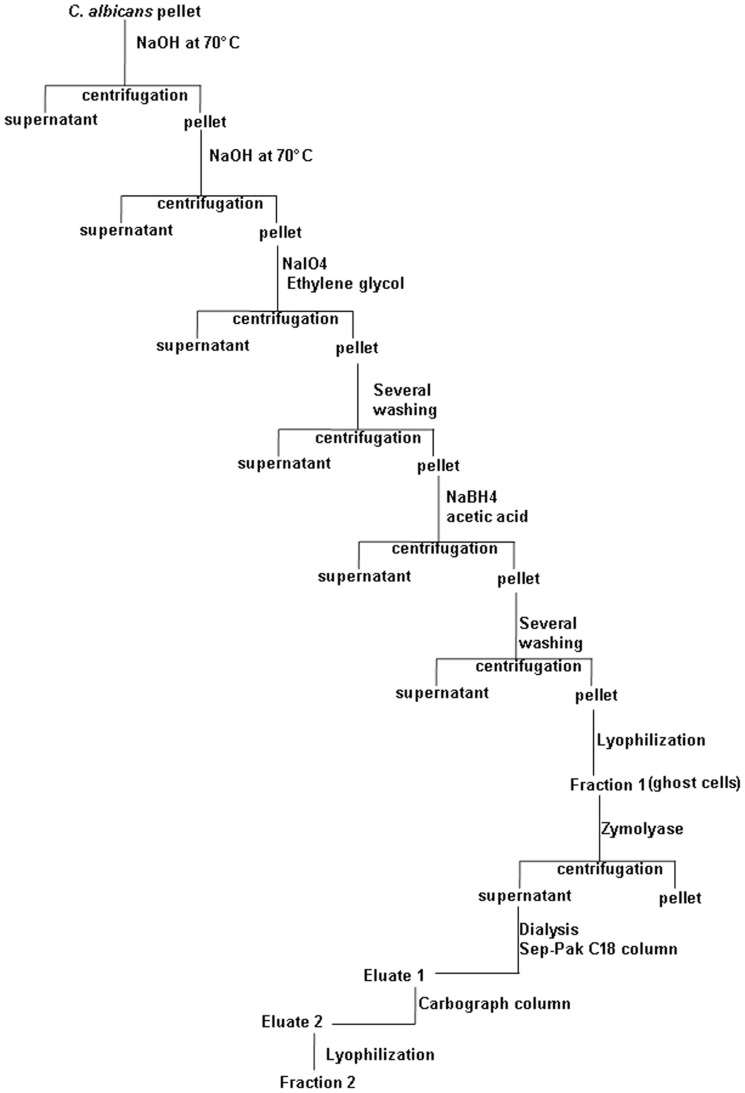
Schematic diagram of β-glucan preparation from *C. albicans*.

**Figure 8:**
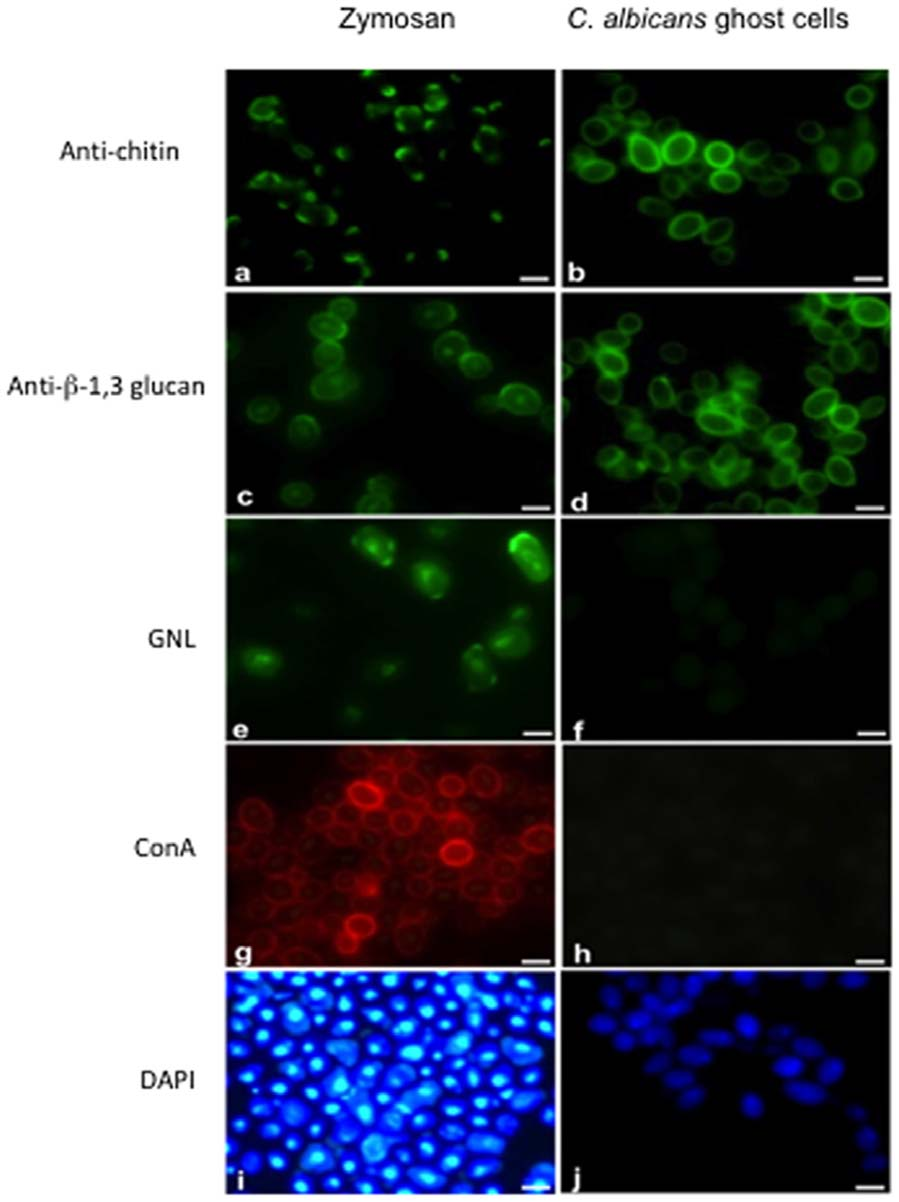
Immunofluorescence staining of *C. albicans* ghost cells and zymosan with various fluorescent probes specific for yeast cell wall glycans. *C. albicans* ghost cells and zymosan were stained with an anti-chitin WGA monoclonal antibody (mAb) (a and b), anti-β-1,3 glucan (c and d), GNL (e and f), ConA (g and h) and DAPI (i and j), respectively. *C. albicans* ghost cells (b and d) and zymosan (a and c) were strongly stained with both anti-chitin WGA and anti-β-1,3 glucan mAb. Strong immunofluorescent staining was detected in zymosan using GNL and ConA (e and g), whereas no immunofluorescent signals were observed in *C. albicans* ghost cells (f and h). While zymosan was strongly stained with nuclear DAPI (i), only a weak signal was observed in *C. albicans* ghost cells (j).

As the glucoprotein fraction from *S. cerevisiae* unexpectedly displayed a protective effect in this model, it was decided to investigate both the structure and biological activity of the β-glucan fraction from *C. albicans*. The harsh whole cell extraction procedure leads to fraction-1, known as yeast ghosts (Fig. 7). Fraction-1 (F1) was analyzed by fluorescence microscopy with various fluorescent probes specific for cell wall glycans in comparison to zymosan, which is widely used for β-glucan immunological studies (Fig. 8). Both zymosan and F1 were labelled with monoclonal antibody (mAb) 2G8 specific for β-1,3 glucans and WGA which binds to chitin (Fig. 8). In contrast to zymosan, which was stained with both Concanavalin A (ConA) and GNL, no mannose residue signals were observed for F1. Immunofluorescent staining with antibodies to β-mannose, liable to be synthesized by *C. albicans*, was also negative with F1 (data not shown). Thus, yeast ghosts have no mannose residues in their cell wall (Fig. 8).

**Figure 9:**
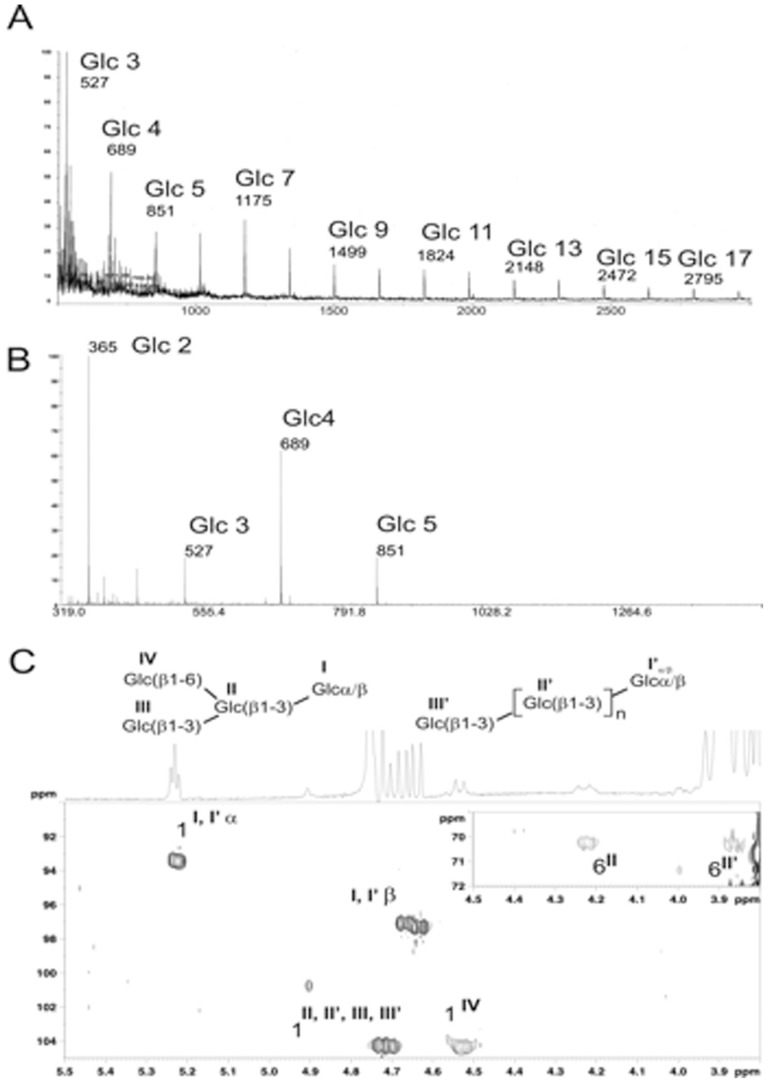
Structural analysis of glycan fraction. (**A**)** MALDI-MS analysis of the native fraction**. Native glycan consisted of a large hexose polymer. (**B**)** Zymolyase digestion of the polymer**. This produced small hexose oligomers, 2–5 residues in size, which were identified by ^1^H-^13^C HSQC-NMR (**C**) as β-1,3-linked glucans partially substituted by single β-glucose residues linked to C-6 positions.

MALDI-MS analysis of the soluble fraction derived from F1 (F2) established that it consisted of a highly polydisperse hexose polymer consisting of 3–27 hexose residues (Fig. 9A). According to its reactivity with anti β-1,3 glucans, this component was susceptible to zymolyase digestion which produced a set of small water soluble fragments (2–5 Glc), as demonstrated by thin-layer chromatography (data not shown) and MALDI-MS analyses (Fig. 9B). After purification of this fraction by reverse phase and adsorption chromatography, its structure was established by NMR as a mixture of β1,3-substituted glucan oligomers with free reducing ends. Furthermore, these oligomers were shown to be partially substituted by a random single β1,6 glucopyranose residue (Fig. 9C).

### Mortality, weight loss, clinical activity, histological score and *C. albicans* colonization

**Figure 10:**
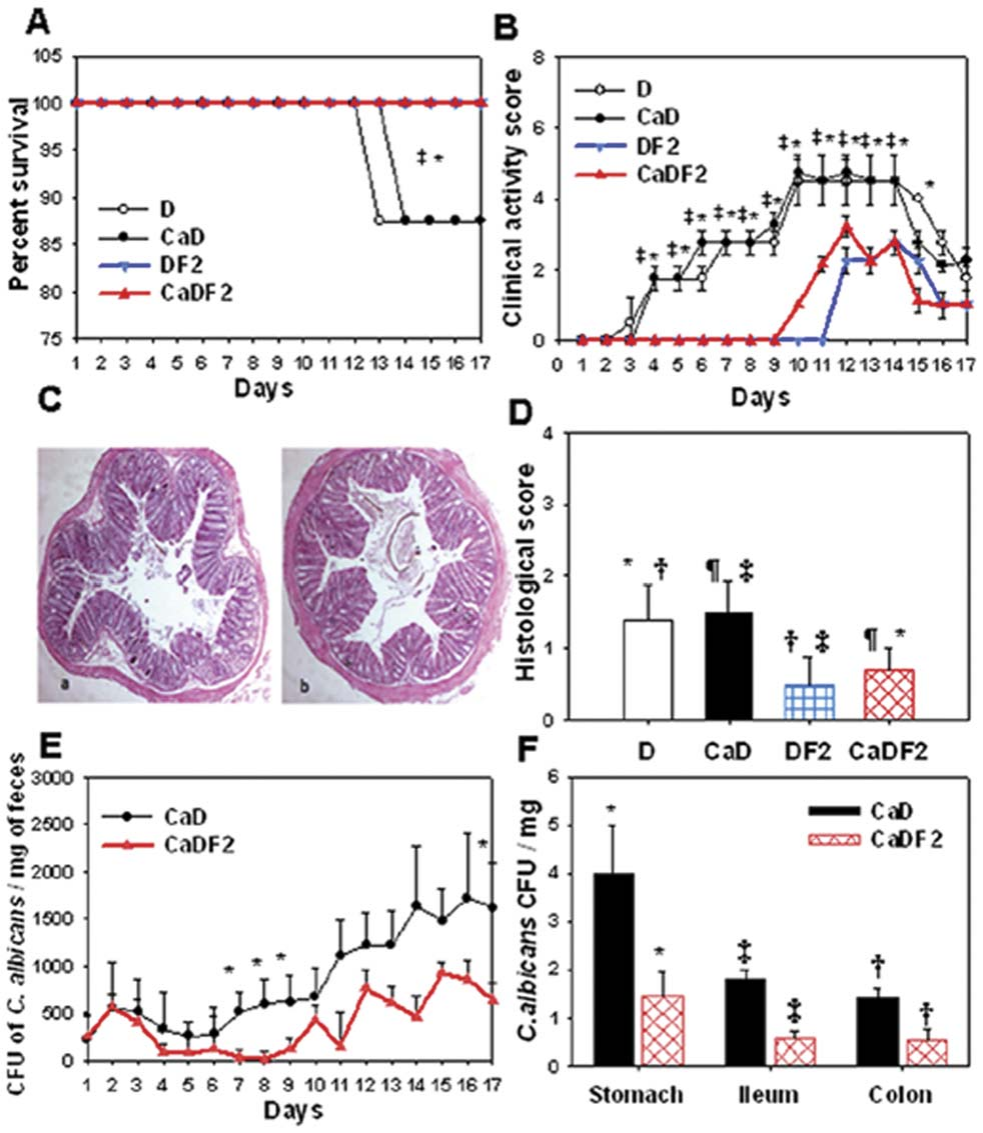
Effect of β-oligoglucoside fractions derived from *C. albicans* on *Candida* DSS-treated mice. (**A**)** Percentage survival of mice**. The results are shown as percent survival from the time of *C. albicans* challenge and DSS treatment. Three days after *C. albicans* challenge, mice were given *C. albicans* β-glucans (F2) for 2 weeks. The survival data were significantly different by the log-rank test. A total of 80 mice were divided into four experimental groups: D (n = 20 mice), CaD (n = 20 mice), DF2 (n = 20 mice), and CaDF2 (n = 20 mice). (*P<0.05 for DF2 mice vs. D mice; and ‡*P*<0.05 for CaDF2 mice vs. CaD mice.) (**B**)** Clinical analysis of DSS-induced colitis in mice**. Each data set represents the mean value for each body weight. (*P<0.05 for DF2 mice vs. D mice; and ‡*P*<0.05 for CaDF2 mice vs. CaD mice.) (**C**)** Histological analysis of DSS-induced colitis in mice**. Panels (a) and (b) correspond to colon sections from DF2 and CaDF2 mice, respectively. No histological signs of colonic inflammation were seen in either DF2 or CaDF2 mice. (**D**)** Histological score**. The histological score decreased significantly in both DF2 and CaDF2 mice. (†*P*<0.05 for DF2 mice vs. D mice; ‡*P*<0.05 for DF2 mice vs. CaD mice; *P<0.05 for CaDF2 mice vs. D mice; and ¶*P*<0.05 for CaDF2 mice vs. CaD mice.) (**E**)** Number of** ***C. albicans*** **colony forming units recovered from stools**. Each data set represents the mean values of eight mice/group. (*P<0.05 for CaDF2 mice vs. CaD mice.) (**F**)** Number of** ***C. albicans*** **colony forming units (CFU) recovered from different gut compartments**. Each data set represents the mean count of 16 mice/group. In mice receiving F2, numbers of *C. albicans* wild-type CFUs from the stomach, ileum and colon were significantly lower than those of *CaD* mice. (*, †, ‡ *P*<0.05 for CaDF2 mice vs. CaD mice.)

After structure characterization of the β-oligoglucoside fraction (F2) extracted from *C. albicans*, the biological activity of F2 was tested in the DSS mouse model. After *C. albicans* challenge, mice were given F2 (1 mg/day) from day3 up to the endpoint (Fig. 1A). F2 administration significantly prevented mouse mortality due to either DSS or DSS+*C. albicans* (Fig. 10A). In contrast to DSS and DSS+*C. albicans* mice, which developed severe colitis and lost body weight, F2 administration reversed the adverse effect of colitis and the mice showed a significant amelioration of both the clinical inflammation score and body weight (Fig. 10B–D). Histological examination of colon sections from mice receiving either DSS or DSS+*C. albicans* showed important colonic inflammation which was associated with mucosal cell loss, crypt damage, mucosal ulceration and accompanying submucosal oedema (Fig. 10C–D). F2 administration significantly reduced colonic inflammation due to either DSS or DSS+*C. albicans* (Fig. 10C–D). *C. albicans* colonization in DSS-treated mice showed a steady increase as assessed by the number of CFUs in faeces which was consistent with the high load of *C. albicans* recovered from the stomach, ileum and colon of this group of mice at the endpoint of the experiments (Fig. 10E–F).

**Figure 11:**
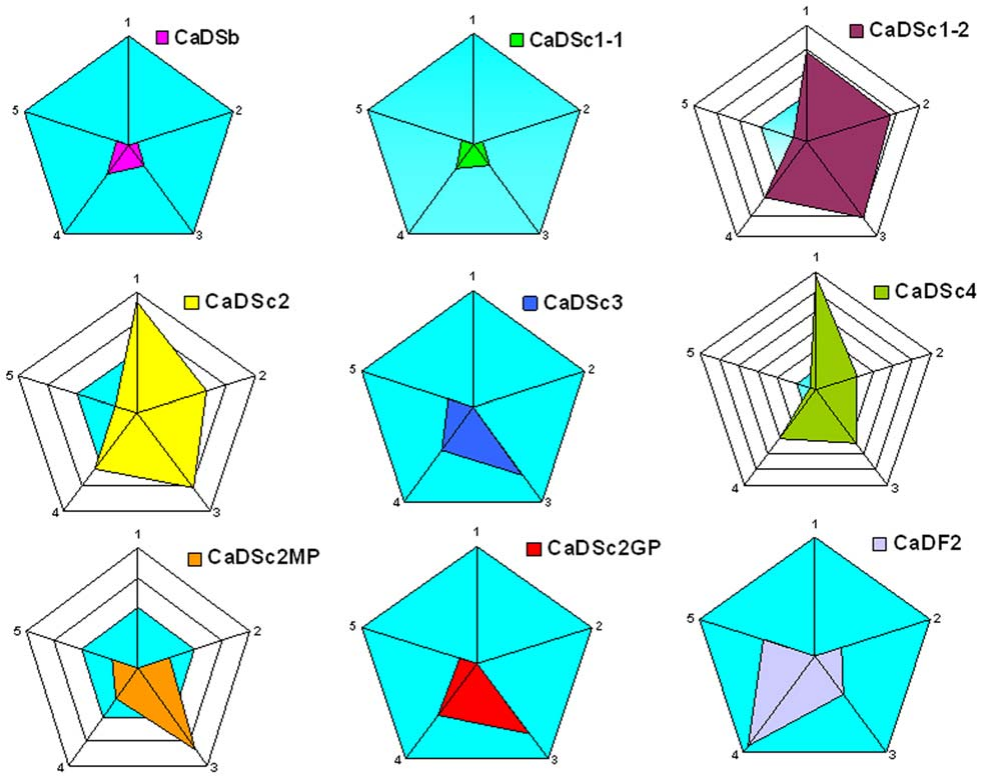
Summary of the effects of *S. cerevisiae* strains or glycan fractions on *Candida* DSS-treated mice. Radar blots showed the effect of each *S. cerevisiae* strain or glycan fraction on: (1) mortality; (2) body weight loss; (3) clinical score; (4) histological score; and (5) *C. albicans* colonization. Sb, Sc1-1 and Sc3 strains improved the intestinal damage due to DSS-induced colitis and *C. albicans* colonization. Sc1-2, Sc2 and Sc4 strains had a dramatic effect on all inflammation signs including *Candida* colonization. Regarding the glycan fractions, both β-glucans and F2 reduced all colitis parameters and eliminated *C. albicans* colonization, while MP was less effective at controlling these parameters.

In contrast to the higher numbers of *C. albicans* CFUs recovered from different compartments of the gut in DSS-treated mice, oral administration of F2 decreased the number of *C. albicans* CFUs recovered from stools and all gut segments of DSS-treated mice (Fig. 10E–F). Furthermore, the reduction in clinical and histological scores was consistent with these low numbers of *C. albicans* CFUs in different parts of the gut (Fig. 11 and Table 2).

**Table 2:** Effect of different strains and cell wall extracts on *C. albicans* colonization and inflammation in the curative *C. albicans* DSS model.

Species/Strain	Mortality at Day 17 (%)	Effect on body weight	Effect on *C. albicans* colonization (CFUs in stools, Day 0–17)	Effect on average clinical scores (Day 0–17)	Effect on histological scores (autopsy or day 17)
		Day 14			
*C. albicans*	12				
Sb	0	+++	+++	+++	+++
Sc1-1	0	+++	+++	+++	+++
Sc1-2	25	−	−	−	−
Sc2	25	−	+	−	+
Sc3	0	+++	+++	++	++
Sc4	75	−	−	−	−
Sc2MP	0	+	−	−	+
Sc2GP	0	++	+++	++	+++
F2	0	++	+++	++	+++

Big improvement (+++), improvement (++), unchanged (+) and worsening symptoms (−).

## Discussion

Excessive use of antifungal agents has been implicated in the emergence of antifungal resistance in *C. albicans* and constitutes a serious clinical problem in hospitals by affecting the natural balance of the intestinal microflora in these individuals [15]. The non-pathogenic yeast, Sb, which is widely prescribed for the treatment of antibiotic-induced gastrointestinal disorders and *Clostridium difficile*-associated enteropathies, has been shown to be an alternative approach to counterbalance the equilibrium of the intestinal microflora and modulate the innate immune defence [16], [17]. Orally administered Sb was successful in both the treatment of inflammatory bowel disease (IBD) and the elimination of *C. albicans* colonization [7], [8], [18]–[21].

Recently, it has been shown that Sb decreases both *C. albicans* colonization and intestinal inflammation in a mouse model of DSS-induced colitis [6]. Following this study, *S. cerevisiae* strains, MP and β-glucan fractions were screened in a mouse model of DSS-induced colitis. As Sb is considered taxonomically to be a strain of *S. cerevisiae* [12], [13], strain Sc1-1 was compared to Sb in the DSS model. Sc1-1 is a gastro-resistant strain that reacts rapidly to its environment and is widely used in the food industry. Incidentally, it was observed that both Sc1-1 and Sb strains reduced *C. albicans* filamentation *in vitro* and *C. albicans* adhesion to plastic-plate wells (data not shown). In the present study, we did not chose a prophylactic but a curative model in which the animals develop colitis with histological features that are similar to those seen in patients with IBD before starting their treatment.

In this model low doses of DSS were used in order to establish *C. albicans* colonization, followed by *S. cerevisiae* or yeast extracts administration to assess their effects on the inflamed colon and colonic epithelium restitution.

Two weeks of DSS administration were scheduled to induce moderate colonic inflammation in mice, with low mortality rates. A recent study by Samonis et al. showed that mice receiving a high daily oral dose of *C. albicans* (around 10^8^ CFU/day) for 2 weeks did not respond to Sb treatment [22]. In our model, a single inoculum of *C. albicans* was used and *Candida* colonization was maintained naturally in the mouse gastrointestinal tract by the DSS-induced colitis since a high *C. albicans* dose could dramatically hide the beneficial effect of Sb. In the present study, and similar to the Sb strain, Sc1-1 decreased both *C. albicans* colonization and intestinal inflammation in terms of clinical and histological score and mortality. Another notable finding was the acceleration of colonic epithelium restoration in mice treated with these dietary yeasts leading to the absence of submucosal oedema and epithelial erosion. Mechanistically, a recent report on intestinal inflammation showed that Sb secretes motogenic factors that enhance intestinal epithelial cell restitution [23].

Regarding the RT-PCR results, both Sb and Sc1-1 reduced the expression levels of pro-inflammatory cytokine TNF-α mRNA in the colonic mucosa with subsequent enhancement of IL-10 mRNA expression that inhibits intestinal injury [24]. Additionally, different pro-inflammatory cytokines were investigated in this set of experiments and were consistent with TNF-α expression. Further investigation is required to determine the role of Th17/Treg responses in different sets of experiments [25], [26]. A recent study in patients with IBD showed that Sb reduced TNF-α production and significantly inhibited T-cell proliferation induced by intestinal inflammation [19]. Generally, the biological activities of *S. cerevisiae* in gastrointestinal inflammatory conditions are mediated through modulation of host pro-inflammatory responses not only by the whole yeast, but also by secreted factors able to interfere with host signalling molecules that control inflammation at different levels such as NF-κB [27], [28]. Sb produces a soluble anti-inflammatory factor that inhibits NF-κB activation and attenuates pro-inflammatory signalling in host cells. In addition, Sb stimulates IL-10 secretion from intraepithelial lymphocytes infected by *C. albicans* and *Escherichia coli* [29]. As Sc1-1 was shown to be comparable to Sb and presents the same beneficial features against *C. albicans* and intestinal inflammation, Sc1-1 was considered as the reference strain in the DSS model. To assess if the observed anti-inflammatory properties were strain-dependent, other *S. cerevisiae* strains were selected deliberately for their high phenotypic diversity. The possible influence of yeast preparation process on anti-*C. albicans* activity was also studied. Surprisingly, some strains had a dramatic effect in the DSS mouse model and the process of yeast preparation also had an influence on the yeast's biological properties [30]. Each strain selected in this study was well characterized *in vitro* in terms of cell growth, osmostress, fermentation, viability and metabolites. However, different factors could influence the biological activity of the strains when introduced by gavage in the DSS mouse model: (i) the resistance of the cell wall related to the yeast preparation process [30]; (ii) the viability of the strain in the stomach, ileum and colon; (iii) its interaction with the microflora and intestinal mucosa [31]; and (iv) its ability to produce soluble anti-inflammatory factors in the milieu triggering expression of mediators in the intestinal epithelium and cells of monocyte lineage present in the submucosae [27]. Altogether, each strain has its own unique properties and supports specific activities within the host. The *in vitro* findings, together with the results for all *S. cerevisiae* strains analyzed in this study, suggest that Sc1-1 has beneficial biological activities reversing all aspects of colitis, including histological damage, diarrhoea and mucosal levels of the pro-inflammatory mediator TNF-α.

The cell wall is an essential structural component of yeast cells playing a central role in the interaction of yeasts with their environment. Unfortunately, the biological activities of *S. cerevisiae* cell wall components are still unclear in terms of *C. albicans* colonization and intestinal inflammation. Two components (MP and β-glucans) produced industrially were explored in our experimental model. With MP fraction administration, *C. albicans* colonization was not consistent with intestinal inflammation parameters, suggesting that MP fractions have differential effects on *C. albicans* colonization and intestinal inflammation. In contrast to MP fractions, GP fraction administration decreased the number of *C. albicans* CFUs concomitantly to all intestinal inflammation parameters.

Both of these components are known to be potent immunological activators, but their mechanisms of action are different and controversial [32]–[34]. As an example, both MP and β-glucans act positively on tumour cells and several microbial infections [34], [35]. Conversely, administration of β-glucans derived from *C. albicans* has been shown to exacerbate arthritis in mice [36]. Structurally, MP have extensive *N*-and *O*-linked mannosylation which serve as ligands for galectin-3 (Gal-3), mannose receptor and DC-SIGN on macrophages and dendritic cells [37]. Different MP express β-Man epitopes, which have been identified as the principal ligand for Gal-3 [38]. In a previous study using the DSS model with *C. albicans*, Gal-3 knock-out mice were less affected by intestinal inflammation and *C. albicans* colonization than wild-type animals [39]. Recently, it was shown that *C. glabrata* deficient in β-Man was less virulent in DSS-treated mice as revealed by low clinical and histological scores and reduction of *C. glabrata* colonization [40]. β-glucans have affinities towards different receptors such as CD11b/CD18 [41], located on neutrophils, or Dectin-1 on macrophages [42]. This results in β-glucan activation of cytokine production and in turn activation of adaptive immunity. Thus, β-glucans attenuate the impact of colitis compared to MP [43].

As our results also showed a beneficial effect of β-glucans on inflammation/colonization, insoluble ghost yeast cells derived from *C. albicans* containing β-glucans were prepared and compared to zymosan which is widely used in β-glucan studies where many investigators refer to it as β-glucan [44], [45]. Zymosan stimulates the production and activity of pro-inflammatory cytokines [45]. Additionally, when chemically characterized zymosan containing only β-(1–3)-glucans was added to macrophage cells, the production of IL-10, reactive oxygen species (ROS) and TNF-α increased in a dose-dependent way [46]. Bonifazi et al. demonstrated the capacity of zymosan to activate both inflammatory and tolerogenic dendritic cells (DCs) leading to the triggering of both Th17 and Treg cells *in vivo* [47]. Our observations showed that zymosan contains both mannans and β-glucans exposed together on the cell wall surface in comparison to *C. albicans* ghosts that contain only β-glucans. This evidence prevented us from further studies on zymosan. Different observations showed that the biological activities of soluble β-glucans differ from those of cell-associated β-glucans [32], [48], [49]. Ishibashi et al. showed that insoluble cell wall β-glucans induced intensive inflammatory and immunomodulating activities compared to soluble β-glucans [49]. Following the β-glucan analysis, the chemical structure of the soluble β-glucan fraction derived from *C. albicans* ghosts was characterized and its biological activities were tested in the DSS mouse model. Interestingly, orally administered β-glucans from *C. albicans* decreased intestinal inflammation and *C. albicans* colonization.Several reports show that β-glucan enhances the immune response and improves the clearance of pathogenic bacteria in animal models [50]–[52]; this supports our findings that smaller oligoglucosides derived from *C. albicans* showed beneficial activities against *C. albicans* and these results are comparable to β-glucans derived from *S. cerevisiae*. However, it may also be hypothesized that these individual oligoglucosides could block receptors such as dectin-1 and CD11b/CD18 and prevent multivalent binding necessary for strong triggering of the inflammatory responses [53]. Besides the importance of yeast molecules sensing for immune response, a third player may also possibly act in the general interplay. This is the mouse microbiota. Oligosaccharides are well known prebiotics active on the intestinal flora [54], [55], and although such a role has not been investigated for *C. albicans* derived oligoglucosides it cannot be ruled out. Altogether, these results demonstrate that oligoglucosides behave differently from the original *C. albicans* whole yeast cells in the DSS mouse model.

In summary, Sc1-1 was found to be comparable to Sb and had beneficial biological activities against *C. albicans* and intestinal inflammation. Clinical trials are currently being conducted with Sc1-1 and promising results have been seen in patients with IBD. In the second part of this study, we focused on cell wall components involved in direct contact with the host and demonstrated that, in contrast to MP, β-glucan fractions from either *S. cerevisiae* or *C. albicans* have a more potent anti-inflammatory effect against colonic colitis induced by DSS in mice. In conclusion, this study generated some progress in deciphering the nature of the yeast molecular components differentially favouring inflammation and/or *C. albicans* clearance. Future studies will include experiments on oligosaccharide administration to mice in order to determine how these glycans stimulate the growth of beneficial bacteria in the gut and boost the immune system providing therapeutic perspectives for digestive disorders and life-threatening fungal infections of endogenous origin.

## Materials and Methods

### Yeast strains

The yeast strains used in this study are shown in Table 1.

### Preparation of β-glucan fractions from yeasts

The composition in dry matter of spray-dried *S. cerevisiae* Sc2 cell wall fractions is shown in Table 3 and the preparation procedure for MP and β-glucan fractions from the cell wall of the same strain is shown in Fig. 1. The fractionation and digestion procedure for extraction of the β-glucan fraction from *C. albicans* is summarized in Fig. 2. Briefly, the cell pellet of *C. albicans* (50 g wet weight) was incubated twice in 200 ml of 1 M NaOH at 70°C for 30 min. After washing with distilled water, the supernatant was removed and the pellet was oxidized with 100 mM NaIO_4_ (Sigma-Aldrich, France) at room temperature for 24 h in the dark [56]. After completion of the reaction, excess periodate was destroyed by adding ethylene glycol. After washing several times with water, the pellet was reduced with 1 M NaBH_4_ (Sigma Aldrich, France) at room temperature. The reaction was terminated by lowering the pH to 5 by the addition of acetic acid. After washing several times with water, the insoluble fraction was then lyophilized to produce fraction-1. Fraction-1 was treated with zymolyase 20T (0.2 mg/mL, Immuno™; ICN Biomedicals Inc.) at 37°C for 3 h. Zymolyase inactivation was performed at 70°C for 5 min. After centrifugation, the supernatant was dialyzed against distilled water. The dialyzed solution was loaded onto a Sep-Pak C18 column (Alltech) equilibrated with 0.1% TFA (trifluoroacetic acid). Eluate-1 was evaporated and the resulting oligoglucosaccharides were dissolved in distilled water and further purified on a carbograph column (Alltech carbograph SPE column). Eluate-2 from the carbograph column was lyophilized to produce fraction-2 (F2). Fluorescence microscopy was performed to assess surface oligomannose expression on fraction-1 in comparison to zymosan (Sigma-Aldrich). Fraction-1 and zymosan suspensions deposited on slides were incubated with either monoclonal antibody (mAb) 2G8 specific for β-1,3 glucans [57], [58], or wheat germ agglutinin(WGA), which binds to chitin [59], or Concanavalin A (ConA) or *Galanthus nivalis* lectin (GNL) or DAPI, as described previously [6], [40]. For animal experimentation, fraction-2 was suspended in water and divided into 200 µL aliquots (each aliquot of 200 µL contained 1 mg of β-glucans).

**Table 3:** Composition in dry matter of spray-dried Sc2 cell wall fractions.

	Proteins	Carbohydrates	Lipids	Minerals
Sc2 MP	48%	42%, comprising 95% β-mannans	Traces	10%
Sc2 GP	5%	60%, comprising 83% glucans85% β-1,315% β-1,6	25%	10%

### Structural analysis of β-glucans

NMR experiments were performed at 300 K using a Bruker AvanceII 900 MHz spectrometer equipped with a 5 mm triple-resonance cryoprobe. Prior to NMR spectroscopic analyses in deuterium, oligosaccharides were repeatedly exchanged in ^2^H_2_O (99.97% ^2^H, Euriso-top; Saint-Aubin, France) with intermediate freeze-drying and finally dissolved in ^2^H_2_O and transferred into Shigemi (Allison Park, USA) tubes. Chemical shifts (ppm) were calibrated taking the methyl group from internal acetone at δ^1^H 2.225 and δ^13^C 31.55 ppm. MALDI-TOF mass spectra were acquired on a Voyager Elite DE-STR mass spectrometer (Perspective Biosystems, Framingham, MA). Prior to analysis, samples were prepared by mixing 1 µL of oligosaccharide solution (1–5 pmol) with 1 µL of 2,5 dihydroxybenzoic acid matrix solution (10 mg/mL in CH_3_OH/H_2_O, 50∶50, vol/vol) directly on the target. Between 50 and 100 scans were averaged for each spectrum.

### Animals

Six- to 8-week-old female BALB/c mice were used. All mice were maintained by Charles River Laboratories (France). Four sets of experiments were performed independently and each experiment was divided into control groups (eight mice/cage), including assessment of the effect of DSS alone, and experimental groups (10 mice/cage).

### Ethics statement

All mouse experiments were performed according to protocols approved by the Subcommittee on Research Animal Care of the Regional Hospital Centre of Lille, France, and in accordance with the European legal and institutional guidelines (86/609/CEE) for the care and use of laboratory animals.

### Inoculum preparation and induction of colitis

Each animal was inoculated on day 1 by oral gavage with 200 µL of phosphate-buffered saline (PBS) containing 10^7^ live *C. albicans* cells. Mice were given 1.5% DSS (MW 36–50 kDa; MP Biomedicals, LLC, Germany) in drinking water from day 1 to day 14 to induce intestinal inflammation. Three days after *C. albicans* oral challenge, mice were administered by oral gavage with a single-daily dose of either 10^7^ lyophilized *S. cerevisiae* strains or 1 mg of β-glucan fraction for 2 weeks. Lyophilized *S. cerevisiae* strains were rehydrated for 30 min in PBS at 37°C before administering to the mice [60]. The presence of yeasts in the intestinal tract was followed daily by performing plate counts of faeces (approximately 0.1 g/sample) collected from each animal [39]. The faecal samples were suspended in 1 mL saline, ground in a glass tissue homogenizer and plated onto Candi-Select medium (Bio-Rad Laboratories, Marnes la Coquette, France). This chromogenic medium is designed for the isolation of yeasts from clinical specimens and is intended to differentiate medically important yeast species depending on the colour of the colonies [61]. Colonies of *C. albicans* were counted after 48 h incubation at 37°C. The results were noted as colony forming units (CFUs)/µg of faeces.

### Presence of *C. albicans* colonization in the gastrointestinal tract

To check for *C. albicans* colonization, the animals were sacrificed and the gastrointestinal tract was removed and separated into the stomach, ileum and colon. The tissues were cut longitudinally. After removal of intestinal contents, the tissues were washed several times in PBS to minimize surface contamination from organisms present in the lumen [62]. Serial dilutions of homogenates were performed. The results were noted as *C. albicans* CFUs/mg of tissue.

### Assessment of clinical parameters

The mortality rate of DSS-treated mice was determined daily and a colon biopsy was taken immediately after death for histological analysis. Total body weight was measured daily. The data are expressed as mean percent change from starting body weight. Daily clinical activity score ranging from 0 to 8 was calculated as described elsewhere [39], [63].

### Determination of histological score

Rings of the transverse part of the colon were fixed overnight in 4% paraformaldehyde-acid and embedded in paraffin for histological analysis. Cross-sections (4 µm thick) were stained with haematoxylin-eosin (Sigma-Aldrich, France). Histological scores were evaluated by two independent, blinded investigators who observed two sections per mouse at magnifications of ×10 and ×100. The scores were determined in accordance with Siegmund et al. [63] and the sections were evaluated for the following two subscores: (i) a score for the presence and confluence of inflammatory cells, including neutrophils, in the lamina propria and submucosa or transmural extension; and (ii) a score for epithelial damage, focal lymphoepithelial lesions, mucosal erosion and/or ulceration and extension to the bowel wall. The two subscores were added together and the combined histological score ranged from 0 (no changes) to 6 (extensive cell infiltration and tissue damage).

### Real-time mRNA quantification

Total RNA was isolated from colon samples using a NucleoSpin RNA II kit (Macherey-Nagel, France) following the manufacturer's instructions, with 20–50 units of DNase I (RNase-free) at 37°C for 30 min to avoid contamination with genomic DNA. RNA quantification was performed by spectrophotometry (Nanodrop; Nyxor Biotech, France). Reverse transcription of mRNA was carried out in a final volume of 26 µL from 1 µg total RNA using 300 U M-MLV reverse transcriptase (Invitrogen, France) according to the manufacturer's instructions with 500 ng oligo(dT) 12–18 and 50 U ribonuclease inhibitor (RNase-Out, Promega). PCR was performed using an ABI 7000 prism sequence detection system (Applied Biosystems, France) with SYBR green (Applied Biosystems, France). Amplification was carried out in a total volume of 25 µL containing 0.5 µL of each primer [6], [39] and 1 µL of cDNA prepared as described above. SYBR green dye intensity was analyzed using Abiprism 7000 SDS software (Applera Corp.). All results were normalized to the housekeeping gene β-actin.

### Statistical analysis

Data are expressed as the mean ± SE of five mice in each group. All comparisons were analyzed by the Mann-Whitney U test. Statistical analyses were performed using the StatView™ 4.5 statistical program (SAS Institute Inc., Meylan, France). Differences were considered significant when the *P* value was <0.05.
